# Complex interactions between malaria and malnutrition: a systematic literature review

**DOI:** 10.1186/s12916-018-1177-5

**Published:** 2018-10-29

**Authors:** D Das, R F Grais, E A Okiro, K Stepniewska, R Mansoor, S van der Kam, D J Terlouw, J Tarning, K I Barnes, P J Guerin

**Affiliations:** 1 WorldWide Antimalarial Resistance Network (WWARN), Oxford, UK; 2 Centre for Tropical Medicine and Global Health, Nuffield Department of Clinical Medicine, University of Oxford, Oxford, UK; 3 Mahidol Oxford Tropical Medicine Research Unit, Faculty of Tropical Medicine, Mahidol University, Bangkok, Thailand; 4 Epicentre, Paris, France; 5 Médecins Sans Frontières, Amsterdam, Netherlands; 6 Liverpool School of Tropical Medicine, Liverpool, UK; 7 Kemri Wellcome Trust Research Programme, Kilifi, Kenya; 8 Division of Clinical Pharmacology, Department of Medicine, University of Cape Town, Cape Town, South Africa; 9 WorldWide Antimalarial Resistance Network (WWARN) Pharmacology, University of Cape Town, Cape Town, South Africa; 10 Malawi-Liverpool Wellcome Trust Clinical Research Programme, Blantyre, Malawi; 11 College of Medicine, University of Malawi, Blantyre, Malawi

**Keywords:** Malaria, Anthropometry, Stunting, Wasting, Underweight

## Abstract

**Background:**

Despite substantial improvement in the control of malaria and decreased prevalence of malnutrition over the past two decades, both conditions remain heavy burdens that cause hundreds of thousands of deaths in children in resource-poor countries every year. Better understanding of the complex interactions between malaria and malnutrition is crucial for optimally targeting interventions where both conditions co-exist. This systematic review aimed to assess the evidence of the interplay between malaria and malnutrition.

**Methods:**

Database searches were conducted in PubMed, Global Health and Cochrane Libraries and articles published in English, French or Spanish between Jan 1980 and Feb 2018 were accessed and screened. The methodological quality of the included studies was assessed using the Newcastle-Ottawa Scale and the risk of bias across studies was assessed using the GRADE approach. The preferred reporting items for systematic reviews and meta-analyses (PRISMA) guideline were followed.

**Results:**

Of 2945 articles screened from databases, a total of 33 articles were identified looking at the association between malnutrition and risk of malaria and/or the impact of malnutrition in antimalarial treatment efficacy. Large methodological heterogeneity of studies precluded conducting meaningful aggregated data meta-analysis. Divergent results were reported on the effect of malnutrition on malaria risk. While no consistent association between risk of malaria and acute malnutrition was found, chronic malnutrition was relatively consistently associated with severity of malaria such as high-density parasitemia and anaemia. Furthermore, there is little information on the effect of malnutrition on therapeutic responses to artemisinin combination therapies (ACTs) and their pharmacokinetic properties in malnourished children in published literature.

**Conclusions:**

The evidence on the effect of malnutrition on malaria risk remains inconclusive. Further analyses using individual patient data could provide an important opportunity to better understand the variability observed in publications by standardising both malaria and nutritional metrics. Our findings highlight the need to improve our understanding of the pharmacodynamics and pharmacokinetics of ACTs in malnourished children. Further clarification on malaria-malnutrition interactions would also serve as a basis for designing future trials and provide an opportunity to optimise antimalarial treatment for this large, vulnerable and neglected population.

**Trial registration:**

PROSPERO CRD42017056934.

**Electronic supplementary material:**

The online version of this article (10.1186/s12916-018-1177-5) contains supplementary material, which is available to authorized users.

## Background

Malaria and malnutrition in children (refers to all forms of undernutrition) are reported independently to be major causes of morbidity and mortality in low- and middle-income countries. About 3.2 billion people remained at risk of malaria with an estimated 216 million new cases (95% confidence interval 196–263 million) and 445,000 deaths worldwide in 2016; the majority of deaths occur in children under 5 years in sub-Saharan Africa [1]. Approximately 3.1 million deaths in children under five are attributed to malnutrition each year, representing 45% of all childhood deaths [2]. The interaction between malaria and childhood malnutrition has been studied for many years and complex interactions between these high-burden conditions are now increasingly recognised. Understanding the consequences of malnutrition on malaria and vice versa is crucial and may help guide the choice of public health interventions and research priorities where significant co-morbidity exists.

Malnutrition is a complex phenomenon due to its multifactorial aetiology and diverse clinical presentation. Acute malnutrition manifests with wasting (low weight for height) and chronic malnutrition as stunting (low height for age). Being underweight (low weight for age) can result from either chronic or acute malnutrition or both. Assessment of malnourishment can be conducted using anthropometric indicators which compare child’s weight and height to the standardised age- and sex-specific growth reference derived from the international reference population of children between 6 and 59 months of age (World Health Organization (WHO) Child Growth Standards 2006) [3]. The anthropometric indicators are expressed as a number of standard deviations (SDs) below or above the reference mean or median value, Z-score. Cutoffs of − 3 are used to indicate severe malnutrition and values between − 2 and − 3 are considered to be moderate malnutrition. Weight-for-height Z-score (WHZ) is the indicator used to classify a child with wasting. Mid-Upper Arm Circumference (MUAC) is another frequently used indicator for wasting. Severe acute malnutrition (SAM) is defined as MUAC < 115 mm and/or WHZ < − 3 and/or bilateral pitting oedema. Stunting is defined by the measure of height-for-age Z-score (HAZ) and a child is considered as being underweight based on low weight-for-age Z-score (WAZ). These definitions do not take micronutrient malnutrition into account, which can occur even if the person is getting enough energy and they are not thin or short.

The exact relationship between childhood malnutrition and malaria remains complex, controversial, and poorly understood. One of the key questions is, to what extent the burden of malaria is attributable to wasting and stunting? In regard to the impact of malaria on malnutrition, some evidence suggests malaria has adverse effects on nutritional status of young children [4–9]. On the other hand, whether and how malnutrition influences malaria morbidity and mortality is debated. Some studies have reported that malnutrition is associated with a higher risk of malaria [10–13], others have suggested a protective effect [14–17], or no differential risk [18, 19]. Similarly, there is very limited evidence on the relationship between nutritional status and antimalarial drug efficacy. Clinical efficacy of the current first-line malaria treatment, the artemisinin combination therapies (ACTs), in malnourished children has been rarely explored [20–22]. Understanding the complex relationship of the immune response of individuals infected with malaria and suffering of malnutrition is crucial to guide specific antimalarial therapeutic approaches in this vulnerable sub-population. There are key knowledge gaps in defining the complex relationship between malnutrition and malaria, which need to be identified and addressed. We aimed to conduct a systematic review of the current understanding of interactions between acute or chronic malnutrition and the risk of developing malarial infection. A further objective was to explore published literature on the impact of malnutrition on the efficacy of antimalarial treatment.

## Methods

We conducted a systematic literature review of manuscripts published between Jan 1, 1980, and Feb 19, 2018. PubMed, Global Health and Cochrane Libraries were searched using key terms (Additional file 1), and articles published in English, French or Spanish were accessed. Two reviewers identified relevant articles of interest, as per criteria listed below, by screening titles and abstracts of publications retrieved. The preferred reporting items for systematic reviews and meta-analyses (PRISMA) guideline [23] was followed. The PRISMA checklist is provided as Additional file 2 and the review is registered in international prospective register of systematic reviews (PROSPERO Registration No. CRD42017056934).

### Eligibility screening

For the selected articles, full text was obtained and assessed for relevance to any of the following topics of interest: (1) association between malnutrition and risk of malaria and (2) malnutrition and antimalarial treatment efficacy. We excluded studies primarily focused on non-malarial conditions such as TB, HIV, neglected tropical diseases, pneumonia or diarrhoeal diseases; non-clinical studies (systematic reviews, opinion pieces, editorials, modelling studies, economic evaluations, guidelines, protocols, book chapters) and in vitro, animal, plant or molecular studies; studies on malaria in pregnancy and placental malaria; demographic and health surveys, mortality surveys, qualitative studies; case reports or series; vaccine studies; and studies primarily focused only on malaria or malnutrition or micronutrient deficiencies or anaemia. For this review, observational and interventional studies in non-pregnant populations with malnutrition assessed by anthropometric measurements as exposure and risk of malaria infection (whether asymptomatic parasitemia or uncomplicated malaria) as outcome were included.

### Data extraction

From each of the included studies in this review, the following variables were extracted: authors, year of publication, country, study design, age (range or median/mean), sex (ratio), sample size, growth standards, malaria transmission intensity, definition of malaria and the reported risk estimates. The methodological quality of the included studies was assessed using the Newcastle-Ottawa Scale (NOS) [24] and the risk of bias across studies was assessed using the GRADE approach [25]. The risk of bias assessment within and across studies is presented as Additional file 3.

### Analysis

Aggregated data meta-analysis was not possible due to the heterogeneity of studies in respect to study design, definition of malnutrition, definition of malaria, study population, e.g. age group target, analysis conducted and effect measures presented. Only summaries of study findings are presented in this review. Association between malnutrition and risk of malaria was deemed to be statistically significant if either the *P* value was < 0.05 and/or the 95% confidence intervals (CIs) did not include 1. The risk of malaria was classified as “increased” or “decreased” according to the interpretation of the effect estimates provided (i.e. incidence risk ratio (IRR), odds ratio (OR), risk ratio (RR) or hazard ratio (HR)) if statistical significance was achieved as described above.

## Results

**Fig. 1:**
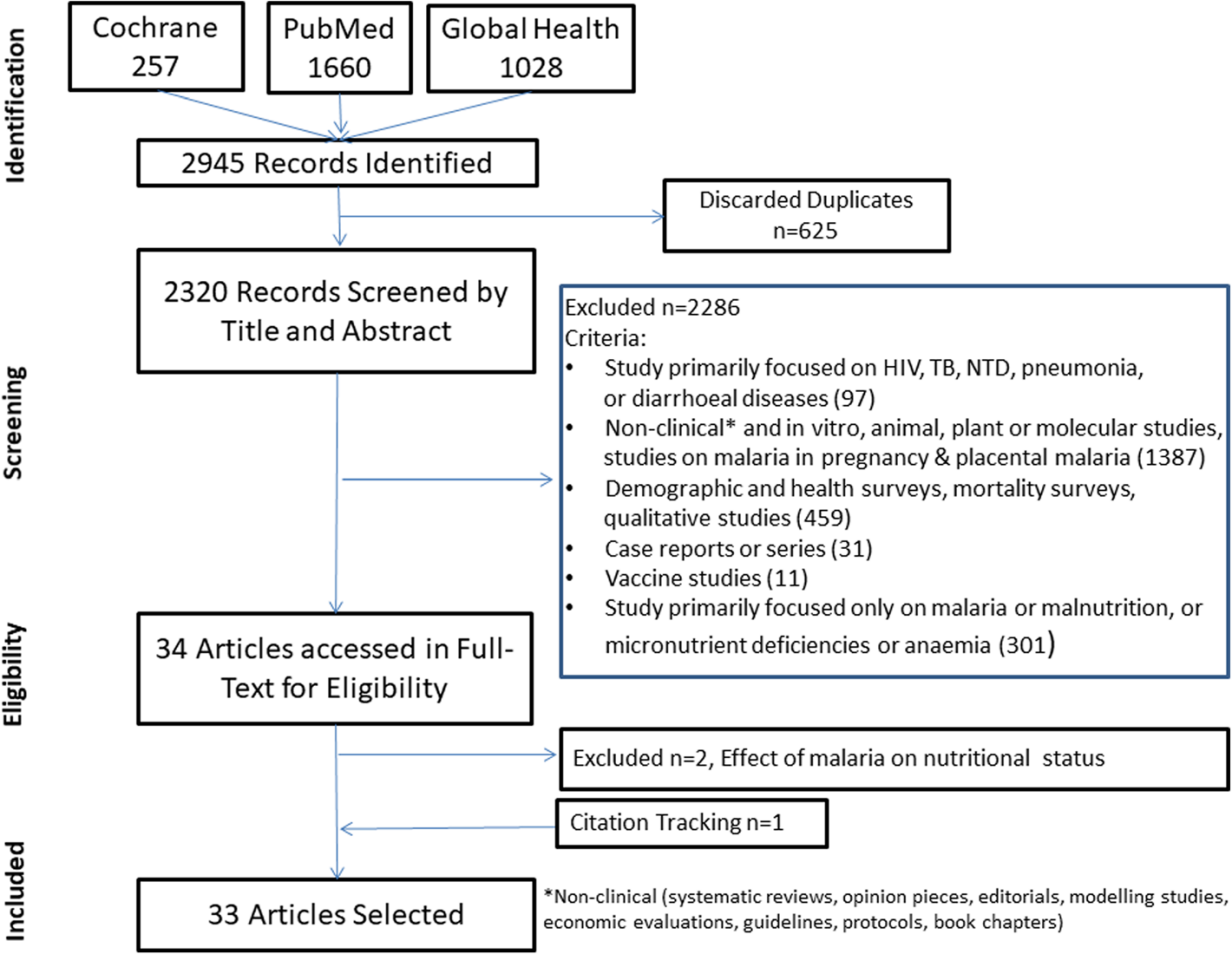
Flow diagram of study selection

The literature search identified 2945 articles. A total of 32 articles identified through the search and 1 article obtained through citation tracking were included, describing cross-sectional surveys (*n* = 16), longitudinal studies (*n* = 12), interventional studies (*n* = 3), case-control study (*n* = 1) and individual patient data meta-analysis (*n* = 1) (Fig. 1). Details of the 33 studies included in this review are given in Table 1, while the study characteristics are summarised in Additional file 4.

**Table 1:** Details of the studies included (*N* = 33)

Author, year, reference	Country	Region	Study design	Topics of interest*
Akiyama 2016 [46]	Lao PDR	South East Asia	Cross-sectional	1a, 1c
Alexandre 2015 [47]	Brazilian Amazon	Latin America	Longitudinal	1a
Arinaitwe 2012 [48]	Uganda	Africa	Longitudinal	1a, 1c
Ayana 2015 [49]	Ethiopia	Africa	Retrospective cohort	1a, 1b, 1c
Bilal Shikur 2016 [28]	Ethiopia	Africa	Case-control	1b
Crookston 2010 [50]	Ghana	Africa	Cross-sectional	1a
Custodio 2009 [30]	Equatorial Guinea	Africa	Survey	1a, 1b, 1c
Deen 2002 [10]	Gambia	Africa	Longitudinal	1a, 1b, 1c
Denoeud-Ndam 2016 [35]	Mali, Niger	Africa	Non-randomised comparative trial	2
Deribew 2010 [51]	Ethiopia	Africa	Cross-sectional	1a, 1b, 1c
Ehrhardt 2006 [11]	Ghana	Africa	Survey	1b, 1c
El Samani 1987 [52]	Sudan	Africa	Cross-sectional	1c
Fillol 2009 [27]	Senegal	Africa	Longitudinal	1a, 1b, 1c
Friedman 2005 [26]	Kenya	Africa	Survey	1a, 1b
Genton 1998 [14]	Papua New Guinea	Oceania	Longitudinal	1a, 1b
Jeremiah 2007 [53]	Nigeria	Africa	Cross-sectional	1c
Kateera 2015 [54]	Rwanda	Africa	Cross-sectional	1a, 1b, 1c
Maketa 2015 [55]	Democratic Republic of Congo (DRC)	Africa	Cross-sectional	1a
Mamiro 2005 [56]	Tanzania	Africa	Cross-sectional	1a
Mitangala 2012 [34]	Democratic Republic of Congo (DRC)	Africa	Therapeutic efficacy study	2
Mitangala 2013 [21]	Democratic Republic of Congo (DRC)	Africa	Survey	1a, 1b, 1c
Muller 2003 [18]	Burkina Faso	Africa	Longitudinal	1a, 1b, 1c
Nyakeriga 2004 [6]	Kenya	Africa	Longitudinal	1a, 1c
Obua 2008 [33]	Uganda	Africa	Therapeutic efficacy study	2
Snow 1991 [17]	Gambia	Africa	Longitudinal	1a, 1b, 1c
Sumbele 2015 [57]	Cameroon	Africa	Cross-sectional	1a, 1b, 1c
Takakura 2001 [29]	Lao PDR	South East Asia	Survey	1b
Tonglet 1999 [32]	Democratic Republic of Congo (DRC)	Africa	Longitudinal	1a, 1c
Uscategui Penuela 2009 [58]	Colombia	Latin America	Cross-sectional	1a, 1b
Verhoef 2002 [13]	Kenya	Africa	Survey	1a, 1b
Verret 2011 [22]	Uganda	Africa	Longitudinal	1a, 1c, 2
William 1997 [31]	Vanuatu	Oceania	Longitudinal	1b, 1c
WWARN Lumefantrine PK/PD Study Group 2015 [36]	Multiple	Multiple	Individual patient data meta-analysis	2

*Topics of Interest: Risk of malaria infection in children with (1a) stunting, (1b) wasting, (1c) underweight; (2) malnutrition and anti-malarial drug efficacy

### Association between malnutrition and risk of malaria

In total, 29 studies assessed the association between malnutrition and risk of malaria (Tables 2, 3 and 4).

**Table 2:** Relationship between chronic malnutrition (stunting) and risk of malarial infection (*N* = 23)

Author, Year, Reference	HAZ cutoff	Malaria outcome	Risk estimate comparing children below and above HAZ cutoff	Risk
Akiyama 2016 [46]	≤ − 2	Asymptomatic malaria confirmed by PCR	OR = 3.34 (95% CI = 1.25–8.93)	Increased
Alexandre 2015 [47]	< − 2	Fever and thick blood smear	HR = 0.31 (95% CI = 0.10–0.99), *P* = 0.049	Decreased
Arinaitwe 2012 [48]	− 1 and −2	Pf malaria, fever and a positive blood smear	IRR = 1.24 (95% CI 1.06–1.46), *P* = 0.008	Increased
	< − 2	Pf malaria, fever and a positive blood smear	IRR = 1.24 (95% CI 1.03–1.48), *P* = 0.02	Increased
Ayana 2015 [49]	< − 2	Malaria by RDT	HR = 2.50 (95% CI = 1.4–5.1)	Increased
Crookston 2010 [50]	≥ − 2 and < − 1	Asymptomatic malaria confirmed by PCR	OR = 2.23 (95% CI = 0.99–5.02)	No impact
	≥ − 3 and < − 2	Asymptomatic malaria confirmed by PCR	OR = 0.56 (95% CI = 0.16–1.69)	No impact
	< − 3	Asymptomatic malaria confirmed by PCR	OR = 1.02 (95% CI = 0.20–3.76)	No impact
Custodio 2009 [30]	< − 2	Pf malaria parasitemia prevalence	OR = 3.07 (95% CI = 1.40–6.73)	Increased
Deen 2002 [10]	< − 2	Malaria episode (fever ≥37.5 °C or parasitemia > 5000/μL)	RR = 1.35 (95% CI = 1.08–1.69), *P* = 0.01	Increased
Deribew 2010 [51]	< − 2	Pf malaria (any parasitemia)	AOR = 0.9 (95% CI = 0.7–1.2), *P* = 0.85	No impact
Fillol 2009 [27]	< − 2	Clinical malaria (fever ≥37.5 °C plus parasitemia ≥ 3000/μL)	“Non-significant association” reported	No impact
	< − 2	High density parasitemia (geometric mean ≥ 300/μL)	AOR = 2.42 (95% CI = 1.12–5.24), *P* = 0.03	Increased
Friedman 2005 [26]	< − 2	Concurrent malaria (any parasitemia)	AOR = 1.98, *P* < 0.0001	Increased
	< − 2	High density parasitemia (any species, > 1500–7000/μL)	AOR = 1.84, *P* < 0.0001	Increased
	< − 2	Clinical malaria (fever plus high density parasitemia)	AOR = 1.77, *P* = 0.06	Increased
	< −2	Severe anaemia (Haemoglobin < 7 g/dL)	AOR = 2.65, *P* < 0.0001*	–
Genton 1998 [14]	< − 2	Pf malaria (fever plus any parasitemia)	Adj. Rate ratio = 1.13 (95% CI = 0.98–1.29), *P* = 0.09	No impact
	< −2	Pf malaria (fever plus parasitemia ≥ 5 × 10^9^/L)	Adj. Rate ratio = 1.19 (95% CI = 1.01–1.40), *P* = 0.03	Increased
	< −2	Pf malaria (fever plus parasitemia ≥ 10 × 10^9^/L)	Adj. Rate ratio = 1.18 (95% CI = 0.98–1.41), *P* = 0.08	No impact
Kateera 2015 [54]	< − 2	Pf malaria (any parasitemia)	“Non-significant association” reported	No impact
Maketa 2015 [55]	≤ − 2	Asymptomatic malaria by blood smear	AOR = 1.8, *P* = 0.01	Increased
Mamiro 2005 [56]	< − 2	Malaria by blood smear	AOR = 1.9 (95% CI = 1.1–3.2), *P* = 0.02	Increased
Mitangala 2013 [21]	< − 2	Pf malaria parasitemia (≥ 5000/μL)	AOR = 0.72 (95% CI = 0.37–1.40)	No impact
	< −3	Pf malaria parasitemia (≥ 5000/μL)	AOR = 0.48 (95% CI = 0.25–0.91)	Decreased
Muller 2003 [18]	≤ − 2	Pf malaria (fever plus parasitemia ≥ 1/μL)	RR = 1.0 (95% CI = 0.9–1.1), *P* = 0.87	No impact
	≤ − 2	Pf malaria (fever plus parasitemia ≥ 5000/μL)	RR = 1.0 (95% CI = 0.9–1.2), *P* = 0.59	No impact
	≤ − 2	Pf malaria (fever plus parasitemia ≥ 100,000/μL)	RR = 0.8 (95% CI = 0.5–1.4), *P* = 0.44	No impact
Nyakeriga 2004 [6]	< − 2	Pf malaria (fever plus any parasitemia)	Adj. IRR = 1.89 (95% CI = 1.01–3.53), *P* = 0.05	Increased
	< − 2	Pf malaria (fever plus any para < 1 year, > 2500/μL > 1 year)	Adj. IRR = 1.93 (95% CI = 0.9–4.16), *P* = 0.09	Increased
Snow 1991 [17]	< − 2	Clinical malaria (fever plus any parasitemia)	“Non-significant association” reported	No impact
	< −2	Asymptomatic malaria parasitemia	“Non-significant association” reported	No impact
Sumbele 2015 [57]	< − 2	Clinical malaria parasitemia	Stunted vs. Non-stunted: 16.9% vs. 7.5%, *P* = 0.01	Increased
	< −2	Asymptomatic malaria parasitemia	Stunted vs. Non-stunted: 26.0% vs. 26.2%, *P* = 0.91	No impact
Tonglet 1999 [32]	< − 2	Clinical malaria without lab confirmation in < 9 m	AOR = 1.16 (95% CI = 0.54–1.77)	Increased
	< −2	Clinical malaria without lab confirmation in ≥ 9 m	AOR = 0.71 (95% CI = 0.28–1.14)	No impact
Uscategui Penuela 2009 [58]	< − 2	Malaria infection	OR = 1.94 (95% CI = 1.07–3.50), *P* = 0.023	Increased
Verhoef 2002 [13]	< −2	Laboratory confirmed malaria	OR = 0.87 (95% CI = 0.69–1.09), *P* = 0.23	No impact
Verret 2011 [22]	≥ − 1 and < 0	Pf malaria risk of recurrent parasitemia	HR = 2.35 (95% CI = 0.85–6.48), *P* = 0.099	No impact
	≥ −2 and < − 1	Pf malaria risk of recurrent parasitemia	HR = 2.89 (95% CI = 1.06–7.89), *P* = 0.039	Increased
	< − 2	Pf malaria risk of recurrent parasitemia	HR = 3.18 (95% CI = 1.18–8.56), *P* = 0.022	Increased

*Pf Plasmodium falciparum*, *HAZ* height-for-age Z-scores, *CI* confidence interval, *OR* odds ratio, *HR* hazard ratio, *RR* risk ratio, *IRR* incidence rate ratio, *AOR* adjusted odds ratio

*Limited to anaemia

**Table 3:** Relationship between acute malnutrition (wasting) and risk of malarial infection (*N* = 18)

Author, Year, Reference	WHZ cutoff	Malaria outcome	Risk estimate comparing children below and above WHZ cutoff	Risk
Ayana 2015 [49]	< − 2	Malaria by RDT	“Non-significant association” reported	No impact
Bilal Shikur 2016 [28]	< − 2	Malaria by RDT or blood film	AOR = 0.66 (95% CI = 0.21–2.03)	No impact
	< − 3	Malaria by RDT or blood film	AOR = 2.90 (95% CI = 1.14–7.61), *P* = 0.025	Increased
Custodio 2009 [30]	< − 2	Pf malaria parasitemia prevalence	“Non-significant association” reported	No impact
Deen 2002 [10]	< − 2	Malaria episode (fever ≥ 37.5 °C or parasitemia > 5000/μL)	RR = 0.87 (95% CI = 0.69–1.10)	No impact
Deribew 2010 [51]	< − 2	Pf malaria (any parasitemia)	AOR = 0.6 (95% CI = 0.2–1.3), *P* = 0.18	No impact
Ehrhardt 2006 [11]	< − 2	Fever	OR = 1.74 (95% CI = 1.16–2.60), *P* = 0.004	.
	< − 2	Clinical malaria (fever ≥ 37.5 °C plus any parasitemia)	OR = 1.86 (95% CI = 1.14–3.02), *P* = 0.007	Increased
Fillol 2009 [27]	< − 2	Clinical malaria (fever ≥ 37.5 °C plus parasitemia ≥ 3000/μL)	OR = 0.33 (95% CI = 0.13–0.81), *P* = 0.02	Decreased
	< − 2	High-density parasitemia (geometric mean ≥ 300/μL)	AOR = 0.48 (95% CI = 0.04–5.34), *P* = 0.55	No impact
Friedman 2005 [26]	< − 2	Concurrent malaria (any parasitemia)	AOR = 0.75, *P* = 0.18	No impact
	< − 2	High-density parasitemia (any species, > 1500–7000/μL)	AOR = 0.96, *P* = 0.88	No impact
	< − 2	Clinical malaria (fever plus high-density parasitemia)	AOR = 1.11, *P* = 0.86	No impact
	< − 2	Severe anaemia (Haemoglobin < 7 g/dL)	AOR = 2.00, *P* = 0.04*	.
Genton 1998 [14]	< − 2	Pf malaria (fever plus any parasitemia)	Adj. rate ratio = 0.92 (95% CI = 0.77–1.11), *P* = 0.4	No impact
	< − 2	Pf malaria (fever plus parasitemia ≥ 5 × 10^9^/L)	Adj. rate ratio = 0.96 (95% CI = 0.77–1.19), *P* = 0.69	No impact
	< − 2	Pf malaria (fever plus parasitemia ≥ 10 × 10^9^/L)	Adj. rate ratio = 0.97 (95% CI = 0.75–1.24), *P* = 0.78	No impact
Kateera 2015 [54]	< − 2	Pf malaria (any parasitemia)	“Non-significant association” reported	No impact
Mitangala 2013 [21]	< − 2	Pf malaria parasitemia (≥ 5000/μL)	AOR = 0.34 (95% CI = 0.08–1.45), *P* = 0.15	No impact
Muller 2003 [18]	< − 2	Pf malaria (fever plus parasitemia ≥ 1/μL)	RR = 1.0 (95% CI = 0.9–1.2), *P* = 0.99	No impact
	< − 2	Pf malaria (fever plus parasitemia ≥ 5000/μL)	RR = 1.0 (95% CI = 0.9–1.2), *P* = 0.58	No impact
	< − 2	Pf malaria (fever plus parasitemia ≥ 100,000/μL)	RR = 1.0 (95% CI = 0.5–1.8), *P* = 0.94	No impact
Snow 1991 [17]	< − 2	Clinical malaria (fever plus any parasitemia)	“Non-significant association” reported	No impact
	< − 2	Asymptomatic malaria parasitemia	“Non-significant association” reported	No impact
Sumbele 2015 [57]	< − 2	Clinical malaria parasitemia	Wasted vs. Non-wasted: 6.5% vs. 9.7%, *P* = 0.78	No impact
	< − 2	Asymptomatic malaria parasitemia	Wasted vs. Non-wasted: 22.6% vs. 26.7%, *P* = 0.77	No impact
Takakura 2001 [29]	< − 2	Pf malaria	Wasted vs. Non-wasted: 17% vs. 4%, *P* < 0.05	Increased
	< − 2	*P. vivax* malaria	“Non-significant association” reported	No impact
Uscategui Penuela 2009 [58]	< − 2	Malaria infection	OR = 2.64 (95% CI = 0.30–23.02), *P* = 0.38	No impact
Verhoef 2002 [13]	< − 2	Laboratory confirmed malaria	OR = 0.78 (95% CI = 0.58–1.05), *P* = 0.1	No impact
William 1997 [31]	< − 2	Clinical malaria (fever plus para ≥ 1000/μL)	“Non-significant association” reported	No impact
	< − 2	*P. vivax* malaria	“Non-significant association” reported	No impact

*Pf Plasmodium falciparum*, *WHZ* weight-for-height Z-scores, *CI* confidence interval, *OR* odds ratio, *HR* hazard ratio, *RR* risk ratio, *IRR* incidence rate ratio, *AOR* adjusted odds ratio

*Limited to anaemia

**Table 4:** Relationship between being underweight and risk of malarial infection (*N* = 19)

Author, year, reference	WAZ cutoff	Malaria outcome	Risk estimate comparing children below and above WAZ cutoff	Risk
Akiyama 2016 [46]	≤ − 2	Asymptomatic malaria confirmed by PCR	OR = 1.33 (95% CI = 0.53–3.30)	No impact
Arinaitwe 2012 [48]	− 1 and −2	Pf malaria, fever and a positive blood smear	IRR = 1.09 (95% CI 0.95–1.25), *P* = 0.24	No impact
	< − 2	Pf malaria, fever and a positive blood smear	IRR = 1.12 (95% CI 0.86–1.46), *P* = 0.39	No impact
Ayana 2015 [49]	< − 2	Malaria by RDT	“Non-significant association” reported	No impact
Custodio 2009 [30]	< − 2	Pf malaria parasitemia prevalence	“Non-significant association” reported	No impact
Deen 2002 [10]	< − 2	Malaria episode (fever ≥ 37.5 °C or parasitemia > 5000/μL)	RR = 1.01 (95% CI = 0.82–1.26)	No impact
Deribew 2010 [51]	< − 2	Pf malaria (any parasitemia)	AOR = 0.9 (95% CI = 0.7–1.2), *P* = 0.90	No impact
Ehrhardt 2006 [11]	< − 2	Fever	AOR = 1.59 (95% CI = 1.13–2.23), *P* = 0.008	Increased
	< − 2	Clinical malaria (fever ≥ 37.5 °C plus any parasitemia)	AOR = 1.67 (95% CI = 1.10–2.50), *P* = 0.009	Increased
	< − 2	Anaemia (Haemoglobin < 11 g/dL)	AOR = 1.68 (95% CI = 1.38–2.04), *P* < 0.0001^φ^	.
El Samani 1987 [52]	Weight-for-age 75–89% (mild)	History of malaria in past 2 months	AOR = 1.20 (95% CI = 0.70–2.00)	No impact
	Weight-for-age < 75% (moderate)	History of malaria in past 2 months	AOR = 2.10, (95% CI = 1.10–4.00)	Increased
Fillol 2009 [27]	< − 2	Clinical malaria (fever ≥ 37.5 °C plus parasitemia ≥ 3000/μL)	“Non-significant association” reported	No impact
	< − 2	High density parasitemia (geometric mean ≥ 300/μL)	AOR = 0.96 (95% CI = 0.35–2.66), *P* = 0.94	No impact
Jeremiah 2007 [53]	< − 2	Malaria by blood smear	RR = 1.02 (95% CI = 0.34–2.37), *P* < 0.02	Increased
Kateera 2015 [54]	< − 2	Pf malaria (any parasitemia)	“Non-significant association” reported	No impact
Mitangala 2013 [21]	< − 2	Pf malaria parasitemia (≥ 5000/μL)	AOR = 0.85 (95% CI = 0.53–1.35), *P* = 0.49	No impact
Muller 2003 [18]	≤ − 2	Pf malaria (fever plus parasitemia ≥ 1/μL)	RR = 1.0 (95% CI = 0.9–1.1), *P* = 0.98	No impact
	≤ − 2	Pf malaria (fever plus parasitemia ≥ 5000/μL)	RR = 1.0 (95% CI = 0.9–1.2), *P* = 0.68	No impact
	≤ − 2	Pf malaria (fever plus parasitemia ≥ 100,000/μL)	RR = 0.8 (95% CI = 0.5–1.4), *P* = 0.49	No impact
Nyakeriga 2004 [6]	< − 2	Pf malaria (fever plus any parasitemia)	IRR = 1.33 (95% CI = 0.64–2.70), *P* = 0.44	No impact
	< − 2	Pf malaria (fever plus any para < 1 year, > 2500/μL > 1 year)	IRR = 0.28 (95% CI = 0.51–3.17), *P* = 0.60	No impact
Snow 1991 [17]	< − 2	Clinical malaria (fever plus any parasitemia)	“Non-significant association” reported	No impact
	< − 2	Asymptomatic malaria parasitemia	“Non-significant association” reported	No impact
Sumbele 2015 [57]	< − 2	Clinical malaria parasitemia	Underweight vs. Non: 21.6% vs. 8.2%, *P* = 0.007	Increased
	< − 2	Asymptomatic malaria parasitemia	Underweight vs. Non: 21.6% vs. 27.5%, *P* = 0.44	No impact
Tonglet 1999 [32]	< − 2	Clinical malaria without lab confirmation in < 9 m	AOR = 1.31 (95% CI = 0.68–1.94)	No impact
	< − 2	Clinical malaria without lab confirmation in ≥ 9 m	AOR = 0.68 (95% CI = 0.24–1.11)	No impact
Verret 2011 [22]	(≥ − 1 and < 0)	Pf malaria risk of recurrent parasitemia	HR = 0.65 (95% CI = 0.37–1.15), *P* = 0.137	No impact
	(≥ − 2 and < − 1)	Pf malaria risk of recurrent parasitemia	HR = 0.86 (95% CI = 0.45–1.62), *P* = 0.636	No impact
	< − 2	Pf malaria risk of recurrent parasitemia	HR = 1.01 (95% CI = 0.54–1.89), *P* = 0.969	No impact
William 1997 [31]	< − 2	Clinical malaria (fever plus parasitemia ≥ 1000/μL)	IRR = 1.1 (95% CI = 0.57–2.1)*, *P* = 0.8	No impact
	< − 2	Clinical malaria (fever plus parasitemia ≥ 1000/μL)	IRR = 1.3 (95% CI = 0.9–1.9)**, *P* = 0.2	No impact
	< − 2	*P. vivax* malaria	IRR = 2.6 (95% CI = 1.5–4.4)*, *P* < 0.0001;	Increased
	< − 2	*P. vivax* malaria	IRR = 1.3 (95% CI = 0.9–2.0)**, *P* = 0.2	No impact

*Pf Plasmodium falciparum*, *WAZ* weight-for-age Z-scores, *CI* confidence interval, *OR* odds ratio, *HR* hazard ratio, *RR* risk ratio, *IRR* incidence rate ratio, *AOR* adjusted odds ratio

^φ^Limited to anaemia;*6 months preceding anthropometric assessment; **6 months following anthropometric assessment

#### Risk of malaria infection in children with stunting (chronic malnutrition)

Twenty-three studies explored the relationship between stunting and risk of malaria infection (Table 2). Overall results were conflicting, with 15 studies showing that stunting was associated with an increased malaria risk, 11 studies showing no association and 2 studies showing a protective effect of stunting (Table 2).

A prospective cohort study of 487 children under 5 years of age in rural Gambia by Deen et al. reported that being stunted increased the risk of malaria infection significantly (RR = 1.35 (95% CI = 1.08–1.69)) [10]. The authors hypothesised that the observed association between malnutrition and malaria infection might be influenced by confounding factors such as HIV co-infection or socio-economic factors. Similarly, in a cross-sectional survey in children < 3 years in Kenya, Friedman and colleagues found an increased malaria risk in stunted children, showing a trend towards an increased risk of clinical malaria (OR = 1.77, *P* = 0.06), and significantly increased risk of any malaria parasitemia (OR = 1.98, *P* < 0.0001), high-density parasitemia (any species, > 1500–7000/μL; OR = 1.84, *P* < 0.0001) and severe anaemia (OR = 2.65, *P* < 0.0001) [26]. In contrast, a longitudinal study from the Gambia by Snow et al. in children aged 1–4 years reported only a minor (non-significant) impact of stunting on clinical and asymptomatic malaria episodes [17]. Two longitudinal malaria surveillance reports, one in Senegal with 874 children aged 12 months–5 years and the other in Burkina Faso with 685 children aged 6–30 months did not show any association between a low HAZ and subsequent malaria attacks [18, 27]. Similarly, Verhoef et al. in Kenya did not observe an association between being stunted and the risk of malaria infection; however, they showed that stunting might determine the severity of malaria-associated anaemia in African children [13]. Verret et al. found that in chronically malnourished children in a high-transmission setting in Uganda, children with mild (HAZ [≥ − 2 and < − 1]) to moderate (HAZ < − 2) stunting not given trimethoprim-sulfamethoxazole prophylaxis were at higher risk for recurrent parasitemia [22]. Contrary to this, in a cohort survey of 790 children under 5 years in the Kivu province, Democratic Republic of Congo (DRC), Mitangala and colleagues found that being severely stunted was protective of subsequent malaria parasitemia [21]. This finding was supported by Genton et al. in a prospective cohort of 136 children aged 10–120 months in Papua New Guinea showing that lower HAZ had a protective effect against *falciparum* malaria [14].

#### Risk of malaria infection in children with wasting

Eighteen studies explored the relationship between wasting and risk of malaria infection (Table 3). Overall, results were again conflicting, with three studies showing that wasting was associated with an increased malaria risk, two studies showing a protective effect and most studies showing no association (Table 3).

Takakura et al. [29], Ehrhardt et al. [11] and Shikur et al. [28] found an increased risk of *P. falciparum* malaria in children with wasting. In a case-control study involving 428 under-five children in Ethiopia, Shikur and colleagues found that severely wasted children were three times more likely to have malaria episode than non-wasted children (adjusted OR = 2.90 (95% CI = 1.14–7.61) [28]. In 2006, Ehrhardt et al. reported a survey involving 2905 children in Ghana aged 6–108 months in which wasting was significantly associated with a higher risk of clinical malaria (OR = 1.86, 95% CI = 1.14–3.02) [11]. Takakura et al. in a cross-sectional study of 309 children and adolescents (aged 2 to 18 years) in the Lao PDR showed that *P. falciparum* infection was associated with wasting [29]. However, Fillol and colleagues reported a significant protective association between being wasted (WHZ < − 2) at the onset of the rainy season and the risk of a clinical malaria episode (OR = 0.33, 95% CI = 0.13–0.81) in 874 preschool children (between 12 months and 5 years of age) in Senegal [27]. Similarly, in a cross-sectional survey of 1862 very young children (from 0 to 36 months age) in western Kenya, Friedman et al. showed that wasting decreased the risk of concurrent malaria (OR = 0.75, *P* = 0.18) and high-density parasitemia (OR = 0.96, *P* = 0.88), although increased the risk of severe malarial anaemia (OR = 2.0, *P* = 0.04) [26]. In contrast, two other longitudinal studies conducted in the Gambia [17] and in Burkina Faso [18] and a few cross-sectional surveys from Equatorial Guinea [30], Eastern Kenya [13] and Ghana [11] reported no association between being wasted and the risk of malaria infections.

#### Risk of malaria infection in underweight children

Nineteen studies explored the relationship between being underweight-for-age and risk of malaria infection (Table 4). Overall, results were again conflicting, with five studies showing that underweight children carried a higher malaria risk, and the remaining studies showing no association (Table 4).

In 2006, Ehrhardt et al. using cross-sectional surveys in Ghana found that being underweight was significantly associated with a higher risk of having fever of any cause (OR = 1.59, 95% CI = 1.13–2.23), clinical malaria (OR = 1.67, 95% CI = 1.10–2.50) and anaemia (OR = 1.68, 95% CI = 1.38–2.04) [11]. This was confirmed by Sumbele et al. [57] who found that 21.6% of underweight children but only 8.2% of adequately nourished children developed clinical malaria (*P* = 0.007) in Cameroon. In a series of cross-sectional surveys conducted in the South Pacific island of Vanuatu in 1997, Williams et al. found a strong association between the incidence of *P. vivax* malaria and subsequently becoming underweight (IRR = 2.6, 95% CI = 1.5–4.4) but no significant effect of *P. falciparum* malaria (IRR = 1.1, 95% CI = 0.57–2.1) [31]. On the other hand, Tonglet et al. reported a non-significant protective association between being underweight and the risk of clinical malaria in children between 9 months and 2 years of age in the DRC (OR = 0.68, 95% CI = 0.24–1.11) [32].

### Malnutrition and anti-malarial drug efficacy

Limited data exist on the effect of malnutrition on response to antimalarial drugs, in particular ACTs. Only five studies were identified in our literature search, and results were again contradictory.

In 2008, Obua et al. explored the impact of nutritional status on the dose, drug concentrations and treatment outcome with co-packaged chloroquine plus sulfadoxine-pyrimethamine in 83 children (6 months–5 years) with uncomplicated falciparum malaria [33]. The authors found that stunting (height-for-age Z-score < − 2) was associated with higher bodyweight-adjusted (mg/kg) doses of chloroquine and sulfadoxine-pyrimethamine, higher sulfadoxine concentrations on day 1 and chloroquine concentrations on day 3, and better cure rates (*P* = 0.046).

In a longitudinal study of 292 infants (aged 4–12 months) in Uganda, a high malaria transmission intensity setting, ACTs (artemether-lumefantrine and dihydroartemisinin-piperaquine) were generally efficacious with a good early parasitological response (99% of study participants cleared parasites by day 3) for treatment of *P. falciparum* malaria, including in 43% chronically malnourished children [22]. However, in this study, stunted children (height-for-age Z-score < − 2) in the dihydroartemisinin-piperaquine arm who were not taking trimethoprim-sulfamethoxazole prophylaxis (given to all HIV-infected and exposed infants) were at higher risk for recurrent parasitaemia (HR 3.18 (95% CI 1.18–8.56); *P* = 0.022). Another study carried out in the DRC in 445 children, comparing the efficacy of standard doses of artesunate-amodiaquine between children with and without severe acute malnutrition (SAM), observed no evidence of reduced efficacy in children with SAM, which had an adequate clinical and parasitological cure rate, ACPR, of 91.4% [34]. A recent multi-centre (Mali and Niger), open-label trial compared the efficacy and pharmacokinetics of artemether-lumefantrine in 399 children with or without SAM. The results of this study showed adequate therapeutic efficacy in both SAM and non-SAM groups (day 42 ACPR 100% vs. 98.3% respectively) with no early treatment failures and no difference in parasite clearance reported. However, a higher risk of reinfection in children older than 21 months suffering from SAM was evident (AHR 2.10 (1.04–4.22); *P* = 0.038) [35]. Similarly in a large pooled analysis of individual pharmacokinetic-pharmacodynamic (PK-PD) data from 2787 patients treated with artemether-lumefantrine for uncomplicated *Pf* malaria, the WorldWide Antimalarial Resistance Network (WWARN) demonstrated that among children 1–4 years of age in high-transmission areas, the risk of reinfection increased with a decrease in WAZ with a HR of 1.63 (95% CI 1.09 to 2.44) for a child with WAZ of − 3 compared to an adequately nourished child (WAZ = 0) [36].

Information on the pharmacokinetic properties of ACTs in malnourished children is critically lacking in the published literature. Our search retrieved a study published in 2016 which assessed the efficacy of AL in relation to drug exposure in children with SAM vs. non-SAM in Mali and Niger [35]. This study measured lumefantrine concentration and showed that despite the administration of 92 g fat with dosing of SAM children (compared to 15 mL milk in non-SAM children), day 7 lumefantrine concentrations were lower in children with SAM compared to non-SAM (median 251 vs. 365 ng/mL, *P* = 0.049). In the WWARN pooled analysis of individual PK-PD data from patients treated with artemether-lumefantrine for uncomplicated *Pf* malaria, underweight-for-age young children (< 3 years) had 23% (95% CI − 1 to 41%) lower day 7 lumefantrine concentrations than adequately nourished children of same age [36].

## Discussion

The evidence on the effect of malnutrition on malaria risk remains controversial and in many instances contradictory. The current review highlights some key limitations in the way the interaction between malaria and malnutrition has been assessed and reported. First, differences in methodology, study populations, the variability in measures used to define malnutrition (e.g. different growth references, different cut off thresholds), and the heterogeneous malaria transmission intensities with different levels of host immunity within the different studies make the comparison challenging. Second, there is a paucity of information on the effect of malnutrition on therapeutic responses to ACTs and their pharmacokinetic properties in malnourished children in published literature. Generally, vulnerable populations with common co-morbidities such as malnutrition, obesity, HIV or tuberculosis co-infection are excluded from or under-represented in antimalarial drug efficacy trials [37]. Although weight is documented, height is rarely recorded in ACT efficacy trials (< 20% of 250 trials currently included in the WWARN repository, personal communication Kasia Stepniewska), restricting the possibilities for secondary analyses. Another useful metric, mid-upper arm circumference (MUAC) is also rarely documented in malaria clinical trials despite being relatively easy to measure and low MUAC shown to be associated with increased malaria risk [38] and decreased lumefantrine bioavailability [39]. Several confounding factors and effect modifiers have been suggested such as age, co-morbidities (e.g. HIV, tuberculosis co-infection and drug interactions), immunity, socio-economic status, or refeeding practices. However, these confounding factors are poorly documented and controlled for in most of the reported studies in this review.

This review has several limitations. First, one third of the studies included in this review recruited individuals of all ages, and disaggregating observations by the age of individuals (below and above 5 years) was not possible. Methodologically, the temporal relationship between malnutrition and risk of malaria (and progression from infection to symptomatic malaria) could not be assessed because of cross-sectional study design (50% included studies). It is also limited by the extent to which important confounders (such as differential micronutrient deficiencies, ecological and genetic factors) are measured and reported in the included articles. Finally, the heterogeneity of the selected studies (presented as Additional file 5) including variations in measurement of nutritional status, definition of malaria, and statistical approaches adopted in deriving the risk estimates restricted plausible aggregated data meta-analysis in this review.

Interestingly, while no consistent association between risk of malaria and acute malnutrition was found, chronic malnutrition was relatively consistently associated with severity of malaria such as high-density parasitemia and anaemia [10, 26, 27]*.* The mechanism behind the higher risk of recurrent parasitemia could be explained partially by the impact of chronic malnutrition on the immune system and/or lower antimalarial bioavailability. Likewise, the apparent protection of wasted children from clinical malaria might be caused by their being administered a higher mg/kg antimalarial dose and/or a modulation of their immune response and thus an absence of symptoms, e.g. fever usually associated with malaria as opposed to an absence of malaria infection. Friedman et al. showed high-density parasitemia as a predictor for chronic malnutrition [26]. Nevertheless, the role of malaria in the aetiology of malnutrition remains unclear. The effect of malaria on nutritional status appeared to be greatest during the first 2 years of life and age acted as an effect modifier in the association between malaria episodes and malnutrition [6].

ACTs are now recommended for the treatment of uncomplicated falciparum malaria in almost all malaria-endemic countries and the number of children exposed to these antimalarial agents is increasing. A priority area is to identify gaps in our current knowledge in regard to the pharmacokinetic properties of artemisinins and partner drugs in malnourished paediatric populations to optimise dosing in order to ensure efficacy, safety and avoid the selection of parasite resistance [40]. Exposing pathogens to sub-therapeutic levels of active ingredients is a major driver of resistance. Protein-energy malnutrition, defined as insufficient calorie and protein intake, may have potential physiological effects on the absorption, distribution and metabolism of ACTs and subsequently affect the efficacy and safety of ACTs. Severe acute malnutrition can cause pathophysiological changes, including increasing total body water, leading to greater volume of distribution of drugs, which in turn may cause sub-optimal drug exposure when ACTs are given at standard doses [35, 41]. This could be further compounded by malnutrition in paediatric patients leading to dosing inaccuracies of ACTs when dose is calculated by age (over-dosing for actual body weight) or weight (under-dosing for age). Malnutrition can also be associated with intestinal malabsorption and villous atrophy of the jejunal mucosa which may cause impaired drug absorption [42]. The reduced absorption of lipids and fats has the potential to specifically affect the lipid-soluble ACTs [43]. The hepatic metabolism of the ACTs may be compromised in malnourished children. For instance, in case of quinine, hepatic metabolism is decreased in protein deficiency and increased in global malnutrition [44]. Thus, hepatic metabolised drugs should be carefully monitored in children with malnutrition. A recent individual patient data (IPD) meta-analysis conducted by the WWARN to investigate the effect of dosing strategy on efficacy of artemether-lumefantrine showed that the risk of treatment failure was greatest in malnourished children aged 1–3 years in Africa (PCR-adjusted efficacy 94.3%, 95% CI 92.3–96.3) [45]. Another large WWARN IPD meta-analysis of individual PK-PD data from patients treated with artemether-lumefantrine (AL) for uncomplicated malaria, showed day 7 concentrations adjusted for mg/kg dose were lowest in very young children (< 3 years), among whom underweight-for-age children had 23% (95% CI − 1 to 41%) lower concentrations than adequately nourished children of the same age and 53% (95% CI 37 to 65%) lower concentrations than adults [36]. This raises the question of whether an adapted dosing regimen is needed in malnourished young children. The PK-PD evaluation of artemisinins and longer-acting partner antimalarials for the treatment of malaria in paediatric populations and the effect of malnutrition on the pharmacological activity of ACTs is a priority area to identify and address key knowledge gaps.

## Conclusions

A summary of the remaining knowledge gaps is presented to serve as the basis for prioritising future research strategies and highlights the need for standardising measures and reporting of nutritional status. Further analyses using individual patient data could provide an important opportunity to better understand the variability observed in publications by standardising both malaria nutritional metrics. In an era of emergence and spread of antimalarial drug resistance, it is imperative to improve our understanding of the pharmacodynamics and pharmacokinetics of ACTs in malnourished children to optimise antimalarial treatment of this very large vulnerable population. Pooled analysis, gap analysis and carefully designed prospective, randomised controlled clinical trials can provide strong evidence on the outstanding questions raised in this review related to malaria-malnutrition interactions.

## Additional files

Additional file 1: PubMed, Global Health, Cochrane database search terms (DOCX 15 kb)

Additional file 2: PRISMA checklist (DOC 67 kb)

Additional file 3: The methodological quality of the included studies (DOCX 33 kb)

Additional file 4: Characteristics of the selected studies (*N* = 33) (XLSX 12 kb)

Additional file 5: Graphical presentation of reported risk estimates by nutritional status (DOCX 135 kb)

## Abbreviations

ACT: Artemisinin combination therapy
AOR: Adjusted odds ratio
CI: Confidence interval
DRC: Democratic Republic of Congo
GRADE: Grading of Recommendations Assessment, Development, and Evaluation
HAZ: Height-for-age Z-score
HR: Hazard ratio
IPD: Individual patient data
IRR: Incidence risk ratio
MUAC: Mid-Upper Arm Circumference
NOS: Newcastle-Ottawa Scale
OR: Odds ratio
Pf: *Plasmodium falciparum*
PK-PD: Pharmacokinetic-pharmacodynamic
RR: Risk ratio
SAM: Severe acute malnutrition
SD: Standard deviation
WAZ: Weight-for-age Z-score
WHO: World Health Organization
WHZ: Weight-for-height Z-score
WWARN: WorldWide Antimalarial Resistance Network
