# Supplementary material for: Complex interactions between malaria and malnutrition: a systematic literature review
Source: BMC Med. 2018 Oct 29;16:186. doi: 10.1186/s12916-018-1177-5 (PMC6205776; doi:10.1186/s12916-018-1177-5)
Supplement: Supplementary file 1 — PubMed, Global Health, Cochrane database search terms (DOCX 15 kb) [file 12916_2018_1177_MOESM1_ESM.docx]

**PubMed, Global Health, Cochrane database search terms**

**PubMed**

(("malaria"[MeSH Terms] OR "malaria"[All Fields]) OR ("plasmodium"[MeSH Terms] OR "plasmodium"[All Fields])) AND (("anthropometry"[MeSH Terms] OR "anthropometry"[All Fields]) OR anthropometric[All Fields] OR ("growth disorders"[MeSH Terms] OR ("growth"[All Fields] AND "disorders"[All Fields]) OR "growth disorders"[All Fields] OR "stunting"[All Fields]) OR ("cachexia"[MeSH Terms] OR "cachexia"[All Fields] OR "wasting"[All Fields]) OR ("thinness"[MeSH Terms] OR "thinness"[All Fields] OR "underweight"[All Fields]) OR ("starvation"[MeSH Terms] OR "starvation"[All Fields]) OR ("protein deficiency"[MeSH Terms] OR ("protein"[All Fields] AND "deficiency"[All Fields]) OR "protein deficiency"[All Fields]) OR ("child nutrition disorders"[MeSH Terms] OR ("child"[All Fields] AND "nutrition"[All Fields] AND "disorders"[All Fields]) OR "child nutrition disorders"[All Fields]) OR ("severe acute malnutrition"[MeSH Terms] OR ("severe"[All Fields] AND "acute"[All Fields] AND "malnutrition"[All Fields]) OR "severe acute malnutrition"[All Fields])) AND ("1980/01/01"[PDAT] : "2018/02/19"[PDAT]) (1660)

**Global Health**

1 exp malaria/ (52575)

2 malaria.af. (71338)

3 exp Plasmodium/ (71245)

4 plasmodium.af. (71771)

5 1 or 2 or 3 or 4 (81080)

6 anthropometric dimensions.sh. (25417)

7 anthropometry.af. (4934)

8 anthropometric.af. (36041)

9 exp growth disorders/ (320)

10 (growth and disorders).af. (27519)

11 stunting.af. (3166)

12 exp cachexia/ (2441)

13 cachexia.af. (1835)

14 wasting.af. (4237)

15 exp thinness/ (262)

16 thinness.af. (1042)

17 underweight.af. (6563)

18 exp starvation/ (3034)

19 starvation.af. (6097)

20 exp protein deficiencies/ (1455)

21 (protein and deficiency).af. (12036)

22 (child and nutrition and disorders).af. (19437)

23 (severe and acute and malnutrition).af. (1047)

24 6 or 7 or 8 or 9 or 10 or 11 or 12 or 13 or 14 or 15 or 16 or 17 or 18 or 19 or 20 or 21 or 22 or 23 (102925)

25 5 and 24 (1077)

26 limit 25 to ((english or french or spanish) and yr="1980 - 2018") (1028)

**Cochrane**

(malaria OR plasmodium) AND ((anthropometry) OR (anthropometric) OR (stunting) OR (wasting) OR (underweight) OR (starvation) OR (Protein Deficiency) OR (Child Nutrition Disorders) OR (Severe Acute Malnutrition)) (257)
