# Supplementary material for: Complex interactions between malaria and malnutrition: a systematic literature review
Source: BMC Med. 2018 Oct 29;16:186. doi: 10.1186/s12916-018-1177-5 (PMC6205776; doi:10.1186/s12916-018-1177-5)
Supplement: Supplementary file 3 — The methodological quality of the included studies (DOCX 33 kb) [file 12916_2018_1177_MOESM3_ESM.docx]

Additional File 3. The methodological quality of the included studies

4a. Risk of bias assessment using Newcastle – Ottawa Scale (NOS): cohort studies

| **Author, Year, Reference** | **Selection** | | | | **Comparability** | | **Outcome** | | | **Total Stars** |
| --- | --- | --- | --- | --- | --- | --- | --- | --- | --- | --- |
|  | Representativeness | Selection of non-exposed cohort | Ascertainment of exposure | Outcome of interest was not present at start of study | Most important factor | Additional factor | Assessment of outcome | Was follow-up long enough for outcomes | Adequacy of follow up of cohorts |  |
| Akiyama 2016 [24] | * | * | * | NA | * |  | * | NA | NA | 5 |
| Alexandre 2015 [25] | * | * | * | * | * |  | * | * |  | 7 |
| Arinaitwe 2012 [26] | * | * | * | * | * |  | * | * |  | 7 |
| Ayana 2015 [27] | * | * | * |  |  |  | * | * |  | 5 |
| Crookston 2010 [29] | * | * | * | NA | * |  | * | NA | NA | 5 |
| Custodio 2009 [30] | * | * | * | NA | * |  | * | NA | NA | 5 |
| Deen 2002 [10] | * | * | * |  | * |  | * | * |  | 6 |
| Denoeud-Ndam 2016 [31] ^$^ | * | * | * |  |  | * | * |  | * | 6 |
| Deribew 2010 [32] | * | * | * | NA | * |  | * | NA | NA | 5 |
| Ehrhardt 2006 [11] | * | * |  | NA | * |  | * | NA | NA | 4 |
| El Samani 1987 [33] | * | * |  | NA | * |  |  | NA | NA | 3 |
| Fillol 2009 [34] | * | * | * | *# | * | * | * | * | * | 9 |
| Friedman 2005 [35] | * | * | * | NA | * |  | * | NA | NA | 5 |
| Genton 1998 [14] | * | * | * | * | * |  |  | * |  | 6 |
| Jeremiah 2007 [36] | * | * |  | NA |  |  | * | NA | NA | 3 |
| Kateera 2015 [37] | * | * | * | NA |  |  | * | NA | NA | 4 |
| Maketa 2015 [38] | * | * | * | NA |  |  | * | NA | NA | 4 |
| Mamiro 2005 [39] | * | * | * | NA |  |  | * | NA | NA | 4 |
| Mitangala 2012 [40] ^$^ | * | * | * |  |  |  | * |  | * | 5 |
| Mitangala 2013 [21] | * | * | * | NA | * |  | * | NA | NA | 5 |
| Muller 2003 [18] | * | * | * | * | * |  | * | * |  | 7 |
| Nyakeriga 2004 [6] | * | * | * |  | * | * | * | * |  | 7 |
| Obua 2008 [41] ^$^ | * | * | * |  |  | * | * |  |  | 5 |
| Snow 1991 [17] | * |  | * |  |  |  | * | * |  | 4 |
| Sumbele 2015 [42] | * | * | * | NA |  |  | * | NA | NA | 4 |
| Takakura 2001 [43] | * | * |  | NA |  |  | * | NA | NA | 3 |
| Tonglet 1999 [44] | * | * | * |  | * |  |  | * | * | 6 |
| Uscategui Penuela 2009 [45] | * |  | * | NA |  |  |  | NA | NA | 2 |
| Verhoef 2002 [13] | * | * | * | NA | * |  | * | NA | NA | 5 |
| Verret 2011 [22] |  | * | * |  | * | * | * |  | * | 6 |
| William 1997 [46] | * | * | * |  |  |  | * | * | * | 6 |

*Star: The NOS tool used a star system to assess the methodological quality based on three criteria; i) participants’ selection (4 stars), ii) comparability of study groups (2 stars), and iii) assessment of outcome/exposure (3 stars). Therefore, the highest total score for a study was 9

NA: Not applicable for cross-sectional study design

$: Interventional studies

#: 27% of study participants were malaria positive at start of the study

4b. Risk of bias assessment using Newcastle – Ottawa Scale (NOS): case-control study

| **Author, Year, Reference** | **Selection** | | | | **Comparability** | | **Exposure** | | | **Total Stars** |
| --- | --- | --- | --- | --- | --- | --- | --- | --- | --- | --- |
|  | Case definition | Representativeness | Selection of control | Definition of control | Most important factor | Additional factor | Ascertainment of exposure | Method of ascertainment | Non-Response rate |  |
| Bilal Shikur 2016 [28] | * | * | * | * | * | * | * |  | * | 8 |

4c. Risk of bias assessment across studies using GRADE approach

| **Quality assessment** |  |  |  |  |  |  |
| --- | --- | --- | --- | --- | --- | --- |
| **No. of studies** | **Design** | **Limitations** | **Inconsistency** | **Indirectness** | **Imprecision** | **Publication bias** |
| **Stunting** |  |  |  |  |  |  |
| N=23 | Cross-sectional 12 | Very serious limitations^a^ | Serious inconsistency^b^ | Not applicable | Serious imprecision^c^ | Unlikely |
|  | Cohort 11 |  |  |  |  |  |
| **Wasting** |  |  |  |  |  |  |
| N=18 | Cross-sectional 10 | Very serious limitations^a^ | Serious inconsistency^b^ | Not applicable | Serious imprecision^c^ | Unlikely |
|  | Cohort 7, Case-control 1 |  |  |  |  |  |
| **Underweight** |  |  |  |  |  |  |
| N=19 | Cross-sectional 9 | Very serious limitations^a^ | Serious inconsistency^b^ | Not applicable | Serious imprecision^c^ | Unlikely |
|  | Cohort 10 |  |  |  |  |  |

^a^Most studies are cross-sectional, temporal relationship between exposure and outcome can’t be assessed

^b^Differences in measurement of nutritional status, definition of malaria and inconsistencies in reported risk estimates

^c^Lack of adjustment in statistical analysis and failure to adequately control confounding factors
