# Supplementary material for: Complex interactions between malaria and malnutrition: a systematic literature review
Source: BMC Med. 2018 Oct 29;16:186. doi: 10.1186/s12916-018-1177-5 (PMC6205776; doi:10.1186/s12916-018-1177-5)
Supplement: Supplementary file 5 — Graphical presentation of reported risk estimates by nutritional status (DOCX 135 kb) [file 12916_2018_1177_MOESM5_ESM.docx]

Additional File 5. Graphical presentation of reported risk estimates by nutritional status


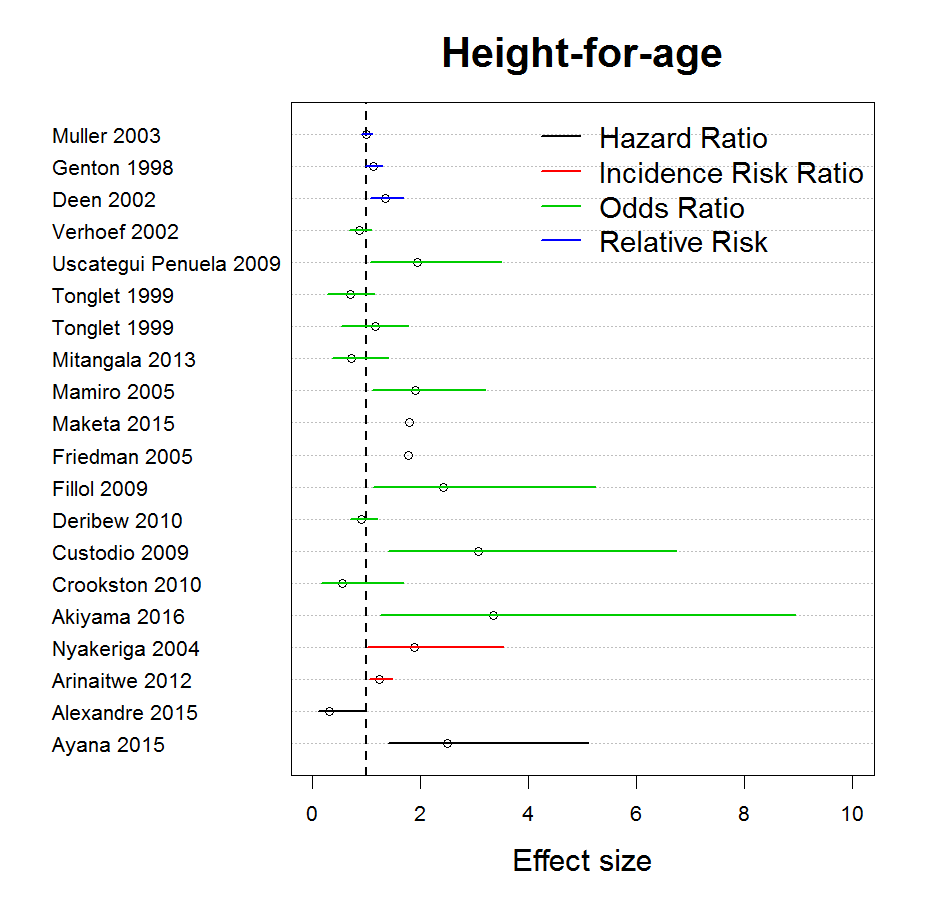


Additional Figure 5a. Risk of malaria infection by stunting status

Legend: Stunting was defined as height-for-age Z-score <-2 or ≤-2. The risk estimates presented are for those who were classified as being stunted as compared to those who were not

Outcomes:

Nyakeriga 2004; Genton 1998; Muller 2003: Pf malaria (fever plus any parasitemia)

Fillol 2009: High density parasitemia (geometric mean ≥ 300/μL)

Friedman 2005: Clinical malaria (fever plus high density parasitemia)

Tonglet 1999: Clinical malaria without lab confirmation in <9 m & ≥ 9 m


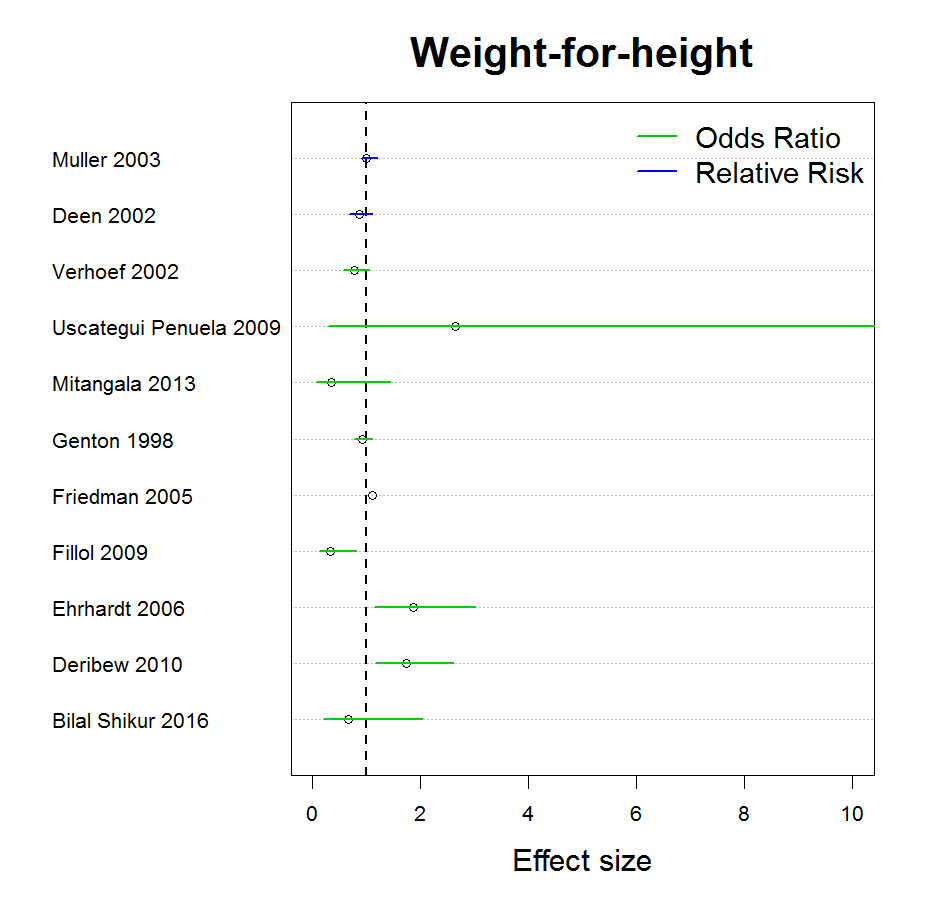


Additional Figure 5b. Risk of malaria infection by wasting status

Legend: Wasting was defined as weight-for-height Z-score <-2. The risk estimates presented are for those who were classified as being wasted as compared to those who were not

Outcomes:

Genton 1998; Muller 2003: Pf malaria (fever plus any parasitemia)

Friedman 2005: Clinical malaria (fever plus high density parasitemia)


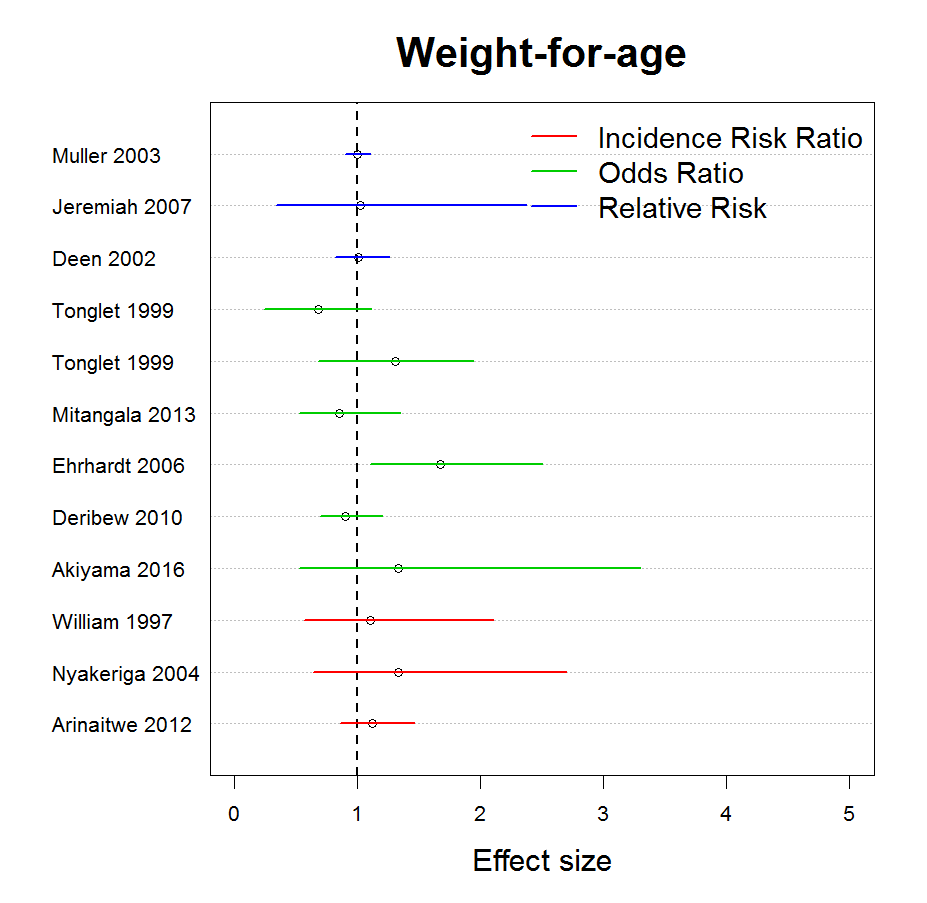


Additional Figure 5c. Risk of malaria infection by underweight status

Legend: Being underweight was defined as weight-for-age Z-score <-2 or ≤-2. The risk estimates presented are for those who were classified as being underweight as compared to those who were not

Outcomes:

Nyakeriga 2004; Muller 2003: Pf malaria (fever plus any parasitemia)

William 1997: Clinical malaria (fever plus parasitemia ≥ 1000/μL)

Ehrhardt 2006: Clinical malaria (fever ≥ 37.5°C plus any parasitemia)

Tonglet 1999: Clinical malaria without lab confirmation in <9 m & ≥ 9 m
